# A case report of bronchial pleomorphic adenoma in a child in China

**DOI:** 10.1186/s12890-020-01338-w

**Published:** 2020-11-11

**Authors:** Haiqin Zhong, Silei Yan, Kung Jiang, Yijing Hu, Xiaoyan Dong

**Affiliations:** Department of Respiratory Medicine, Children’s Hospital of Shanghai, Shanghai Jiao Tong University, Shanghai, China

**Keywords:** Pleomorphic adenoma, Bronchus, Child, Case report

## Abstract

**Background:**

Paediatric cases of pleomorphic adenoma of the bronchus are rare in clinical practice, despite pleomorphic adenoma being the most common histological form of salivary gland neoplasm. To date, no such cases have been reported in China.

**Case presentation:**

We report a case of pleomorphic adenoma of the bronchus in a 10-year-old child with no obvious positive signs on examination. Chest-enhanced computed tomography and bronchoscopy showed a large white mass in the right principal bronchus. The patient was treated by bronchial mass resection. Biopsy confirmed the diagnosis of pleomorphic adenoma.

**Conclusions:**

We not only describe a rare benign bronchial tumour in children but also demonstrate the successful use of surgery as a radical cure for pleomorphic adenoma.

## Background

Pleomorphic adenoma, also called mixed tumour, salivary gland type, is the most common benign tumour of the salivary glands, most frequently occurring in the parotid gland (approximately 80%), followed by the jaws, submandibular gland, sublingual gland, cheeks, mouth, and lips [1]. Paediatric cases of pleomorphic adenoma of the bronchus are rare in clinical practice and are easily missed during diagnosis and misdiagnosed due to their slow growth and nonobvious symptoms in the early stage [2]. To date, no such cases have been reported in China. Due to the rarity of intrabronchial pleomorphic adenoma, no formal study has described its treatment or provided long-term follow-up results. The main treatments include surgical resection and bronchoscopic interventional therapy. We report a case of bronchial pleomorphic adenoma, which was successfully removed by surgery.

## Case presentation

**Fig. 1:**
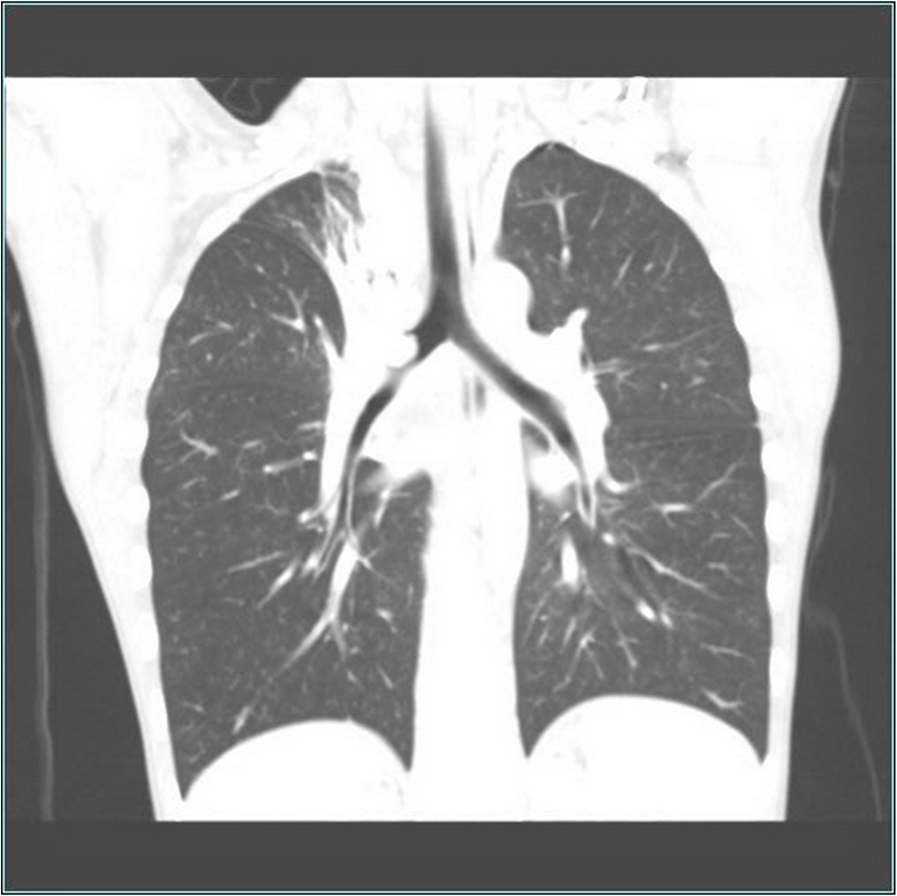
Chest CT shows a space-occupying lesion in the right principal bronchus

**Fig. 2:**
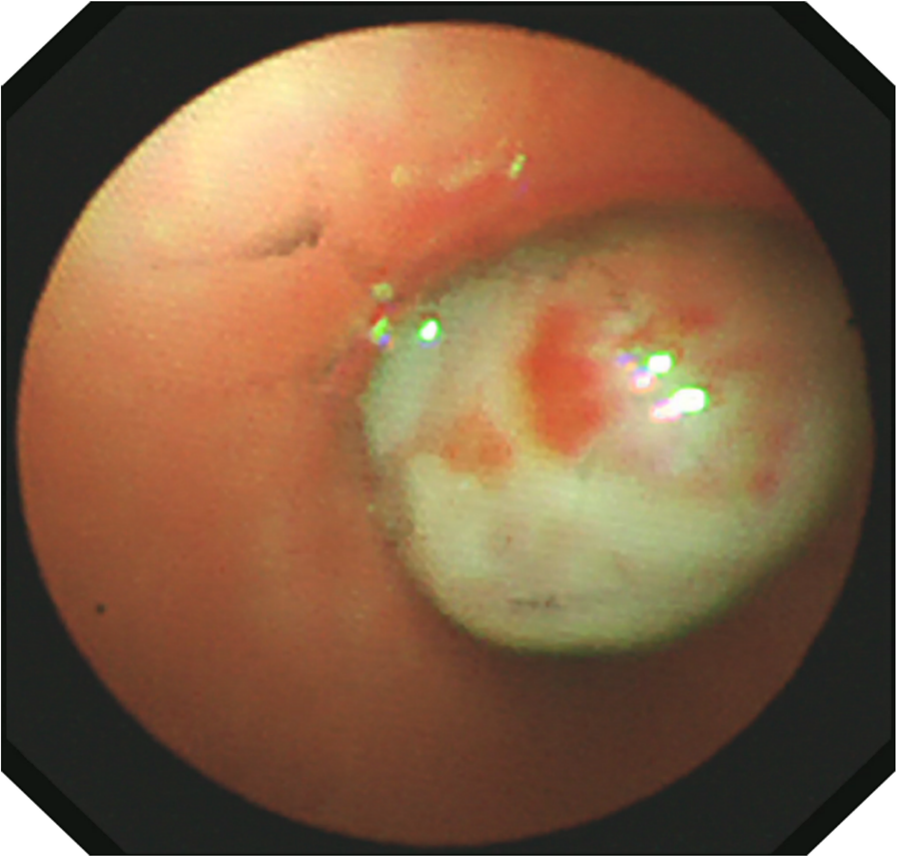
A mass was observed under bronchoscopy obstructing the lumen in the right principal bronchus

**Fig. 3:**
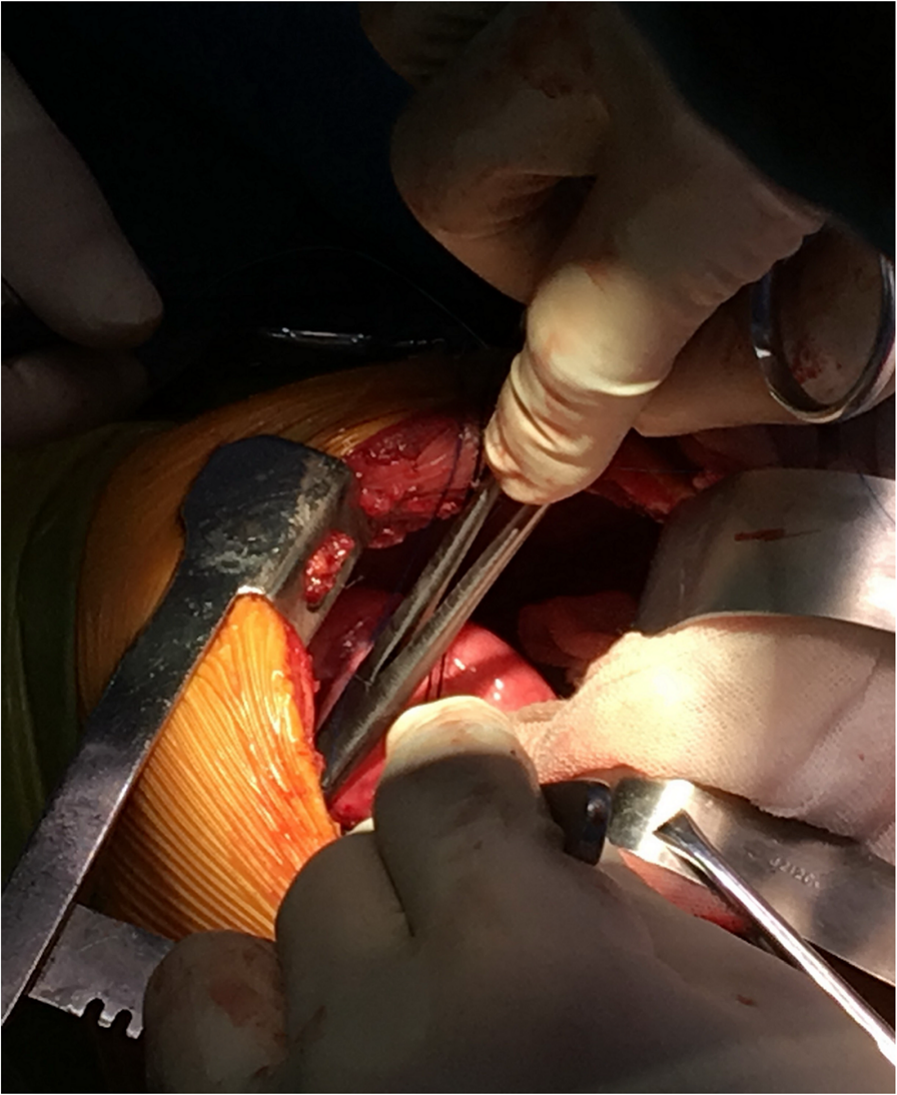
Bronchial mass resection and right upper lobectomy

A 10-year-old boy was admitted to the other hospital because of coughing for 7 days 1 month ago and had undergone a chest computed tomography scan. The scan revealed a 1.3-cm mass occupying the right main bronchus and atelectasis in the upper lobe of the right lung. Bronchoscopy and biopsy were performed 12 days after the computed tomography scan. Bronchoscopy showed hyperplasic tissue in the right main bronchus. Pathology showed incisional hypersensitivity and hyperplasia of the right bronchus. Chronic inflammation of the bronchial mucosa was observed. The epithelial cells of the mucosa were proliferated and squamous, with some papillary hyperplasia. After the child was admitted to our hospital, no obvious positive signs were observed during physical examination. An enhanced computer tomographic scan was conducted, which showed a mass occupying the right main bronchus and obstructive pneumonia with atelectasis, as shown in Fig. 1. We also performed another bronchoscopy, which showed that the right principal bronchus was almost completely blocked by a large white mass, as shown in Fig. 2. On the 10th day of admission, “bronchial mass resection and extended resection” was performed in the Department of Thoracic Surgery, as shown in Fig. 3.

**Fig. 4:**
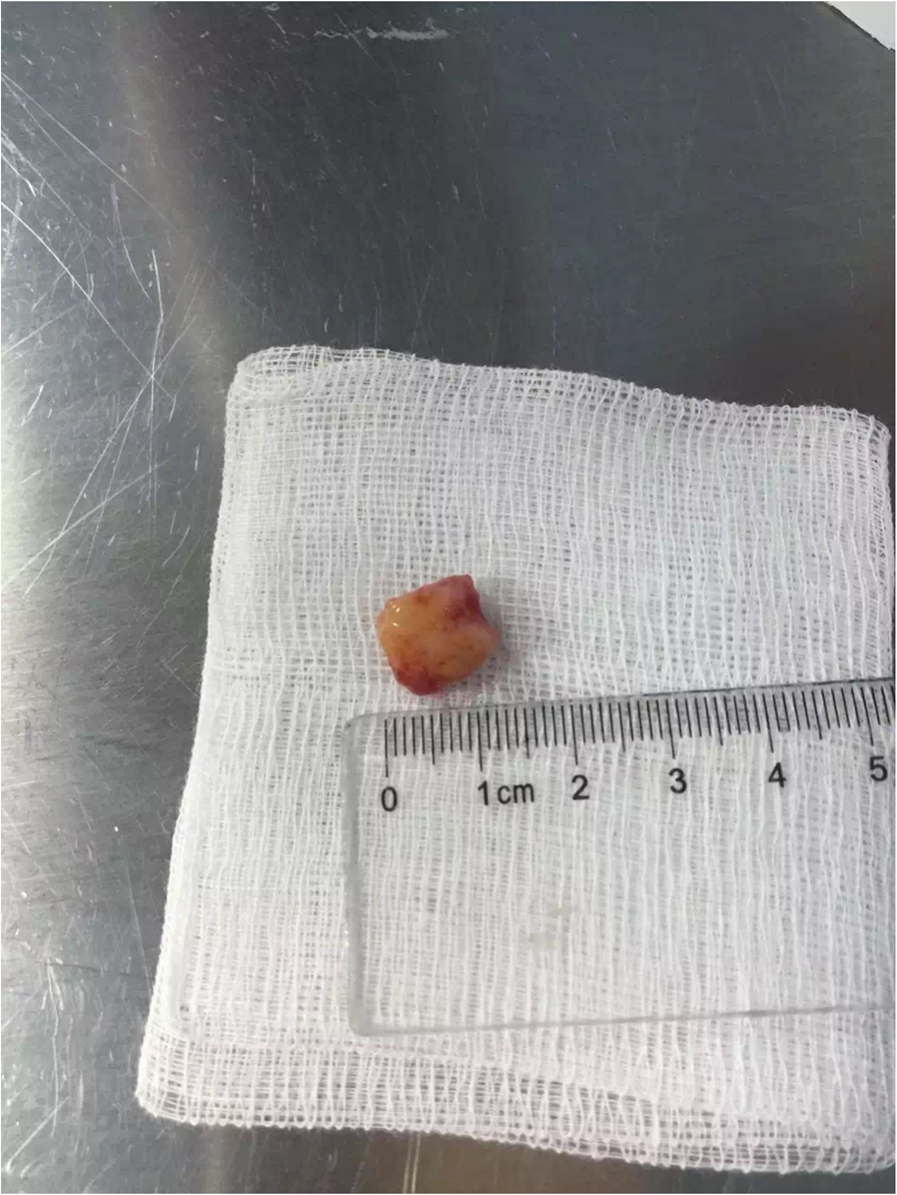
The bronchial mass

**Fig. 5:**
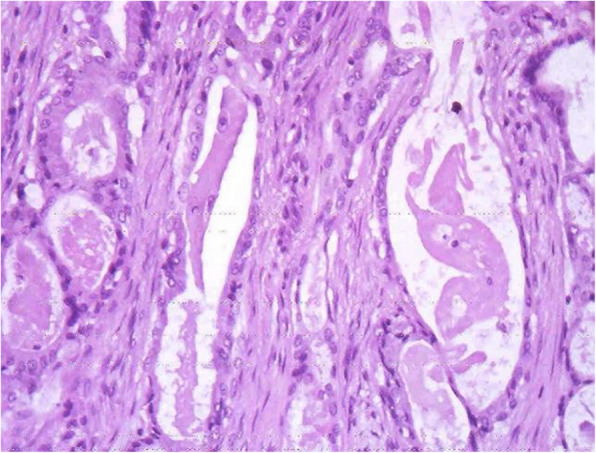
Tissue morphology under a pleomorphic adenoma light microscope (HE, × 200)

**Fig. 6:**
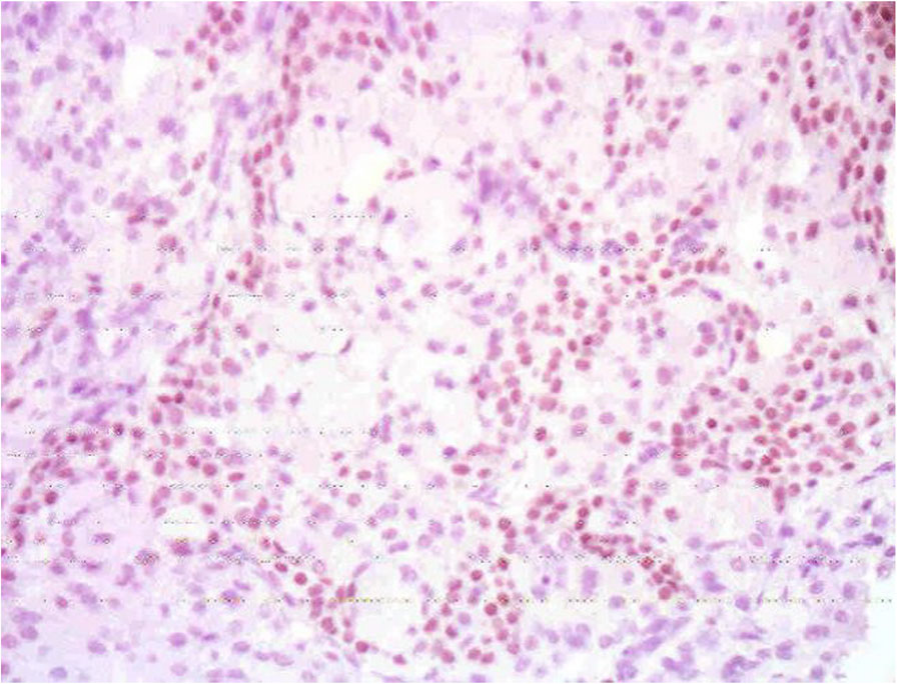
P63 positive cells in tumour tissues (En Vision, × 200)

Examination of gross specimens obtained during the operation showed a piece of grey-white tissue with a volume of 1.1 cm × 0.7 cm × 0.6 cm and a smooth surface with a medium texture, as shown in Fig. 4. Light microscopy showed that the tumour consisted of a mixture of glands, tubules, cysts, and solid regions, dominated by glandular components and covered by columnar mucous cells, goblet cells or cubic clear cells. Tumour tissues of solid nests or pieces and sections consisted of basal-like and intermediate-type cells and, small numbers of squamous cells. Immunohistochemistry results: 1. Vimentin ++, 2. CAM5.2 ++, 3. EMA ++, 4. P63 ++, 5. CD99 ++, 6. HHF35 ++, and 7. Ki-67 5% ± (proliferation index). Pathological diagnosis: pleomorphic adenoma (as shown in Fig. 5 and Fig. 6). The child was generally in good condition after the operation and was discharged from the hospital after recovery. No recurrence or tumour metastasis was found at the 1-month follow-up.

## Discussion and conclusions

Pleomorphic adenoma is characterized by a complex and diverse morphological structure, many mucocartilage-like regions with thin, incomplete or no envelopes, the features of borderline tumours, and a certain likelihood of malignant transformation. In recent years, studies have found a certain recurrence rate of pleomorphic adenoma after surgery that is closely related to carcinogenesis [3]. Based on reported cases, the incidence of bronchial pleomorphic adenoma is highest in adults, while cases in children are rare [4].

Relevant literature regarding tracheal and bronchial pleomorphic adenoma was searched from 2000 to 2019, and twenty-eight cases have been reported in total, including 15 males and 13 females. One case of a child under 18 years old was found [5], 3 cases occurred in patients aged 18 to 30 years [6, 7], 13 cases occurred in patients aged 31 to 50 years [8–20], and 11 cases occurred in patients older than 50 years [21–31]. Of the 12 cases from foreign countries, 4 were in Japan [17, 28–30], 3 were in Turkey [15, 18, 27], 2 were in South Korea [20, 31], 1 was in Malaysia [16], 1 was in Iran [5], and 1 was in Mexico [19]. Thus, 27 cases were in Asia, while 1 was in North America, suggesting that pleomorphic adenoma mainly occurs in the Asian population. Our case involved a 10-year-old child, and this article represents the first report of bronchial pleomorphic adenoma in China. Cough, expectoration and dyspnoea were the most common clinical manifestations.

The mechanism of pleomorphic adenoma is still unclear, and pleomorphic adenoma must be differentiated from mucoepidermoid carcinoma, carcinoid, and hamartoma during diagnosis. Squamous metaplasia may present in pleomorphic adenoma; a few sebaceous gland cells or mucous goblet cells appear in the squamous cell cluster of the metaplasia, which is easily misdiagnosed as mucoepidermoid carcinoma. However, the metaplasia does not contain myoepithelial cells, and intermediate cells are found in mucoepidermoid carcinoma with abundant mucus cell and negative myoepithelial cell labelling, which can aid in distinguishing between the two tumour types in diagnosis [32]. Carcinoid tumours derived from the Kulchitisky cells in the bronchial mucosa are a kind of neuroendocrine carcinoma that mainly occur in the bronchus of adults. In addition to the asthma caused by obstruction of the respiratory tract, varying degrees of haemoptysis may be present, and a few patients may have symptoms of carcinoid syndrome. Currently, due to the lack of relevant research, the pathogenesis of bronchial pleomorphic adenoma in children is not completely clear. Therefore, the wheezing and dyspnoea caused by bronchial pleomorphic adenoma are difficult to distinguish from non-occupying diseases such as bronchial asthma and chronic bronchitis due to the hidden onset and slow growth of pleomorphic adenoma, leading to high rates of misdiagnosis and missed diagnosis. The resulting damage to the lung tissues poses a severe threat to the health and safety of affected children.

In the case presented in this report, pleomorphic adenoma occurred in the bronchus of a paediatric patient. No similar reports have been released in China. Due to the rare occurrence of this condition, the treatment includes interventional endoscopy and surgery but has not been standardized for various cases. Pleomorphic adenoma is mostly benign, with slow growth and, no obvious signs or specific symptoms in the early stage, and is difficult to diagnose, especially in hidden areas of the human body such as the bronchus, which often delays early diagnosis and treatment. Due to the large volume of the tumour in the late stage of growth, it can easily apply pressure on the bronchus and surrounding lung tissue, leading to lung tissue damage. In this case, the patient presented with chronic inflammation and hyperplasic of the bronchial mucosa in the right main bronchus. Pathology showed hyperplasia and squamatization of epithelial cells and, some papillary hyperplasia. Baghai-Wadji et al. [5] reported that children with bronchial pleomorphic adenoma present with recurrent infections of cysts and diffuse pneumonia in the lung tissue. Lung lobectomy was performed for clinical treatment of the children. The damage to lung tissue caused by bronchial pleomorphic adenoma requires further study.

Bronchial pleomorphic adenoma is generally a solid or translucently colloidal circular or round mass with a clear boundary, a complete or incomplete envelope, and a greyish-white or grey-yellow surface on sections. The tumour consists of epithelial and interstitial components. Many morphological features are evident in the pathology, and heterogeneity exists between different types of tumours, substantially complicating diagnosis only by fine needle aspiration or small-sample biopsy.

Although bronchial pleomorphic adenoma is a benign tumour, malignant transformation can occur, and complete surgical resection is the best treatment. However, the type of surgery recommended depends on the individual characteristics of the patient and the size and location of the tumour. Resection should be conducted 0.5 cm–1 cm away from the tumour cells, and tumour rapture should be avoided at all costs. For patients who cannot undergo surgery, interventional therapy via bronchoscopy is the recommended course of action. However, bronchoscopic interventional therapy can only remove intracavitary masses; negative incisal edges cannot be guaranteed, and residual tumour tissue may persist. This therapy serves only as a palliative and temporary treatment of the acute airway obstruction.

Pleomorphic adenoma is characterized by multicentricity, multiple occurrences, an incomplete envelope, tumour infiltration, and growth breaking through the envelope, which are the common reasons for postoperative recurrence. Pathological studies have shown that residual tumour cells or tumour cell seeding after rupture of the envelope is the main cause of the high recurrence rate after surgery [33, 34]. Therefore, patients with pleomorphic adenoma should undergo thorough and complete resection, and the scope of resection should be expanded if necessary. In this case, the child presented with a typical case of pleomorphic adenoma. After undergoing extended resection in our hospital, the patient recovered well, and follow-up observations will be carried out.

In summary, pleomorphic adenoma, especially bronchial pleomorphic adenoma, has an insidious onset, and cases in children are rare both locally and abroad. The diagnosis can be confirmed using bronchoscopy and postoperative biopsy. Preoperative examinations for children with bronchial pleomorphic adenoma should be improved to verify the anatomy of the tumour and the state of the lung tissue such that an appropriate surgical method can be selected to improve the prognosis.

## Data Availability

The datasets used and/or analyzed during the current study are available from the corresponding author on reasonable request.
